# Effect of Blue Light and Photosensitizers on *Cutibacterium acnes* on Shoulder Periprosthetic Joint Infection Isolates

**DOI:** 10.7150/jbji.46199

**Published:** 2020-06-20

**Authors:** Swati Bhargava, Thomas Listopadzki, Sara Diletti, John K. Crane, Thomas R. Duquin, K. Keely Boyle

**Affiliations:** 1 Department Medicine, Division of Infectious Disease, University at Buffalo, Buffalo, NY.; 2 Jacobs School of Medicine and Biomedical Sciences, University at Buffalo, Buffalo, NY.; 3 Department of Orthopaedics, State University of New York at Buffalo, Buffalo, NY.

**Keywords:** *Propionibacterium acnes*, shoulder infection, demeclocycline, anaerobic pathogens, fluorescein, riboflavin

## Abstract

**Introduction:** *Cutibacterium acnes* is gaining recognition as a leading pathogen after orthopaedic shoulder procedures. Photodynamic therapy, a combination of light and a photosensitizer, has demonstrated antimicrobial activity against *C. acnes* in the treatment of acne vulgaris. We sought to evaluate the effect of photodynamic therapy using blue light and photosensitizers on *C. acnes* isolates from shoulder prosthetic joint infections.

**Methods:** *C. acnes* strains isolated from 19 patients with shoulder PJI were exposed to blue light alone (415 nm) or in combination with photosensitizers (fluorescein, riboflavin and demeclocycline). *C. acnes* strains were divided into 4 categories: *Highly Sensitive (HS), Sensitive (S)*, *Weakly Sensitive (WS), Resistant* to blue light.

**Results:** 13 of 19 *C. acnes* strains (68%*)* were *S* or *HS* to blue light alone. Of these 19 strains tested, 11 were tested with blue light and fluorescein or blue light plus riboflavin. Fluorescein (1 µg/mL) enhanced the effect of blue light in 6 of 11 strains (55%). Blue light plus riboflavin (10 µg/mL) resulted enhanced killing in 3 of 11 strains (27%), but produced a paradoxical photoprotective effect in 4 of 11 strains (36%), resulting in a net decrease compared to blue light alone. Demeclocycline, however, enhanced the effect of blue light in 16 of 17 strains (94 %).

**Conclusions:** Blue light with the addition of photosensitizers killed *C. acnes* from periprosthetic shoulder infections *in vitro*, with demeclocycline having the most pronounced effect.

## Introduction

*Cutibacterium acnes (C. acnes)* is recognized as one of the leading infecting pathogens after orthopaedic shoulder surgeries, especially arthroplasty procedures 1, 2. Infection caused by *C. acnes*, formerly* Propionibacterium acnes,* presents unique diagnostic and therapeutic challenges to orthopaedic surgeons as the patient's clinical presentation is usually indolent and diagnostic markers may be within normal limits. Additionally, positive cultures during the infection workup could be dismissed as contamination due to the bacterium's pervasive presence in human skin flora 3-9. Several factors predispose a patient to infection, including endogenous host factors, skin surface topography, balance of milieu of microorganisms, and exogenous environmental factors 10.

Current preoperative measures for prevention of orthopaedic shoulder infections include administration of preoperative antibiotics, skin decolonization methods, meticulous soft tissue handling, hemostasis and appropriate wound closure 11. Previous studies have shown *C. acnes* strains to be very susceptible to first generation cephalosporins, specifically cefazolin, which is the most commonly utilized perioperative antibiotic for joint replacement procedures in the United States 12-14. Current skin decolonization protocols for joint replacement procedures include chlorhexidine gluconate or iodine-based solutions 15, 16. More recently proposed infection prevention techniques include the use of preoperative topical benzoyl peroxide or topical clindamycin 8, 17-19. Despite these efforts, *C. acnes* can persist and cause periprosthetic joint infections (PJI), which necessitates the development of novel infection prevention strategies 20.

Photodynamic therapy (PDT) involves the use of light sensitive molecules called photosensitizers in combination with an ultraviolet or visible light source. Light of a particular wavelength activates the photosensitizer, which then reacts with oxygen to produce reactive oxygen species leading to cell death 21-23. PDT has also shown encouraging results against a variety of pathogens including Gram-negative infections (*Pseudomonas aeruginosa* and *Acinetobacter baumannii)*, Gram-positive infections (*C. acnes*, methicillin-sensitive *Staphylococcus aureus* (MSSA), methicillin-resistant *Staphylococcus aureus* (MRSA), *Staphylococcus epidermidis),* as well as other pathogens (fungi, viruses, parasites) implicated in skin infections, wounds, dental infections, and implant-related biofilm disruption 5, 23, 24.

Prior studies have shown that light-based therapies, including blue light, may inhibit *C. acnes* growth associated with acne vulgaris 25, 26. Blue light is an attractive light source due to its broad-spectrum antimicrobial effect and greater safety in mammalian cells compared to ultraviolet light therapy. We sought to evaluate the effect of the PDT using blue light and photosensitizers on *C. acnes* strains from periprosthetic shoulder infections.

## Materials and Methods

### Blue light source

The source of blue light was the Omnilux Clear-U light-emitting diode (LED; Photo Therapeutics, Carlsbad, CA). This light source emits blue light with peak emission at 415 nm, and includes a built-in cooling system so that heat is dissipated. Light energy output, or fluences, delivered at 15, 30, 45 and 60 minutes were reported to be 17.5 J/cm^2^, 35 J/cm^2^, 52.5 J/cm^2^ and 70 J/cm^2^ 24. Using 415 nm light, the Absorbance of Luria-Bertani (LB) broth was 0.177 and that of thioglycollate broth was 0.486, meaning that blue light absorption is substantial in microbiological media.

### Bacterial strains used

A total of 19 isolates were carefully selected from a collection of *C*.* acnes* strains isolated from Musculoskeletal Infection Society (MSIS) determined shoulder PJIs. Of these strains, 9 were from the collection of Crane et al. 12 and 10 were from the collection reported by Wright et al. 27.

### Photosensitizers

#### Fluorescein

Fluorescein was chosen due its low toxicity and FDA approved status for use in the eye and intravenously 28. Fluorescein has a very high molar absorptivity at the wavelength of ~488 nm and the large fluorescence yield and high photostability have made this a common fluorescent label in various applications of medicine 24. The concentration of fluorescein used was 1 µg/mL and was based on concentrations reported in prior substantiated literature and as refined during the initial phases of this study.

#### Riboflavin

Riboflavin (Vitamin B_2_) was also chosen as a candidate photosensitizer because of its low toxicity, solubility in water, and reported antimicrobial activity 29. The absorbance spectrum spans a larger wavelength from ~310 nm to 700 nm, with a peak occurring ~440 nm 30. Varying concentrations of riboflavin have been utilized depending on the outcome measure, sought after efficacy and clinical application 29, 31. The concentration of riboflavin used was 10 µg/mL and was based on concentrations reported previously for varying applications and also refined during the initial phases of this study.

#### Demeclocycline

Demeclocycline was chosen because, unlike riboflavin and fluorescein, it is a semi-synthetic tetracycline antibiotic that also functions as a photosensitizer. While other tetracyclines absorb ultraviolet (UV) light, demeclocycline can be effectively activated by blue light (415 nm) and UV light with an absorption peak occurring at ~366 nm 32. The demeclocycline concentrations used, 1.5-2.5 µg/mL, were chosen to be within achievable serum concentrations in humans, and kept as low as possible in order to limit the direct antibiotic effect on the *C. acnes* in the absence of blue light 33.

### C. acnes Susceptibility to Tetracycline Antibiotics

Since demeclocycline is an uncommonly used antibiotic, there is no commercial supplier of MIC strips or E-test strips for demeclocycline. Therefore, we determined the MIC of demeclocycline using the agar dilution method, using brain-heart infusion (BHI) agar, as described by Wang et al. 34. The range of demeclocycline concentrations tested was from 0.5 to 4 mg/mL. In order to be able to compare the results of demeclocycline with doxycycline, we also used BHI agar for the doxycycline MIC strip testing. Susceptibility to doxycycline was determined using doxycycline MIC strips (Liofilchem USA, Waltham, MA).

Determination of susceptibility breakpoints to antibiotics for *C. acnes* is difficult. Older versions of the breakpoint tables for *C. acnes* compiled by the Clinical Laboratory Standards Institute (CLSI) and the European Committee on Antimicrobial Susceptibility Testing (EUCAST) used to include some interpretive breakpoints, although the breakpoints were often classified as tentative 5. In the most recent update, EUCAST susceptibility tables contain no antibiotic breakpoints for *C. acnes*. Nevertheless, based on our research, we adopted as a cut-off a doxycycline MIC or 1.0 µg/mL or greater as being resistant to doxycycline (Table 1).

### Blue Light Exposure

The *C. acnes* strains were grown in tubes of thioglycollate medium, enriched with hemin and vitamin K, until visible growth was observed (Hardy Diagnostics; Santa Maria, CA & Anaerobe Systems; Morgan Hill, CA). Optical Density at 600 nm (OD_600_) was measured using a SmartSpec3000 spectrophotometer (Bio-Rad, Carlsbad, CA). The bacteria were diluted into sterile normal saline to obtain culture turbidity equivalent to a 0.5 McFarland standard (OD_600_ of ~ 0.1 to 0.15). After dilution, the bacterial density was approximately 10^7^ or 10^8^ CFU/mL. Photosensitizers were added, and then bacterial suspensions were allowed to warm to 37 º for 5 minutes on a heater block before placement in the 96 well plate(s) (see Flow Diagram in Supplemental Material).

Diluted bacterial suspensions were divided and tested under 4 conditions: zero blue light (dark control), blue light alone (No Additive), and blue light with photosensitizers, as well as blue light plus ethanol vehicle. An ethanol vehicle control was tested for the photosensitizers that had to be dissolved in ethanol (fluorescein, demeclocycline). Testing the solvent alone was performed to make sure the effect being studied was not due to the solvent used, in this case, 0.05- 0.1% ethanol (Table 2). Aliquots of 250 µL of the diluted bacterial suspensions were placed into wells of a flat-bottomed 96-well plate in room air at 37º C, beginning with the samples to be exposed the longest (45 or 60 min).

Blue light was placed directly on top of the 96-well plate, approximately 0.5 cm from the surface of the liquid. Aliquots of bacterial suspension were transferred from the dark to the illuminated plate at 15 min intervals. Of note, the suspensions that were kept in the dark were exposed to the photosensitizer for the full duration of the experiment, allowing us to determine if there was any effect of the photosensitizer alone, without light (i.e., dark toxicity).

After calculated exposure, irradiated bacterial suspensions were diluted using serial 10-fold dilutions in sterile normal saline. Subsequently, 3 µL aliquots from each well were spotted onto Brucella Blood Agar plates (Hardy Diagnostics, Santa Maria, CA) to quantitate survival after blue light exposure.

**Figure 1:**
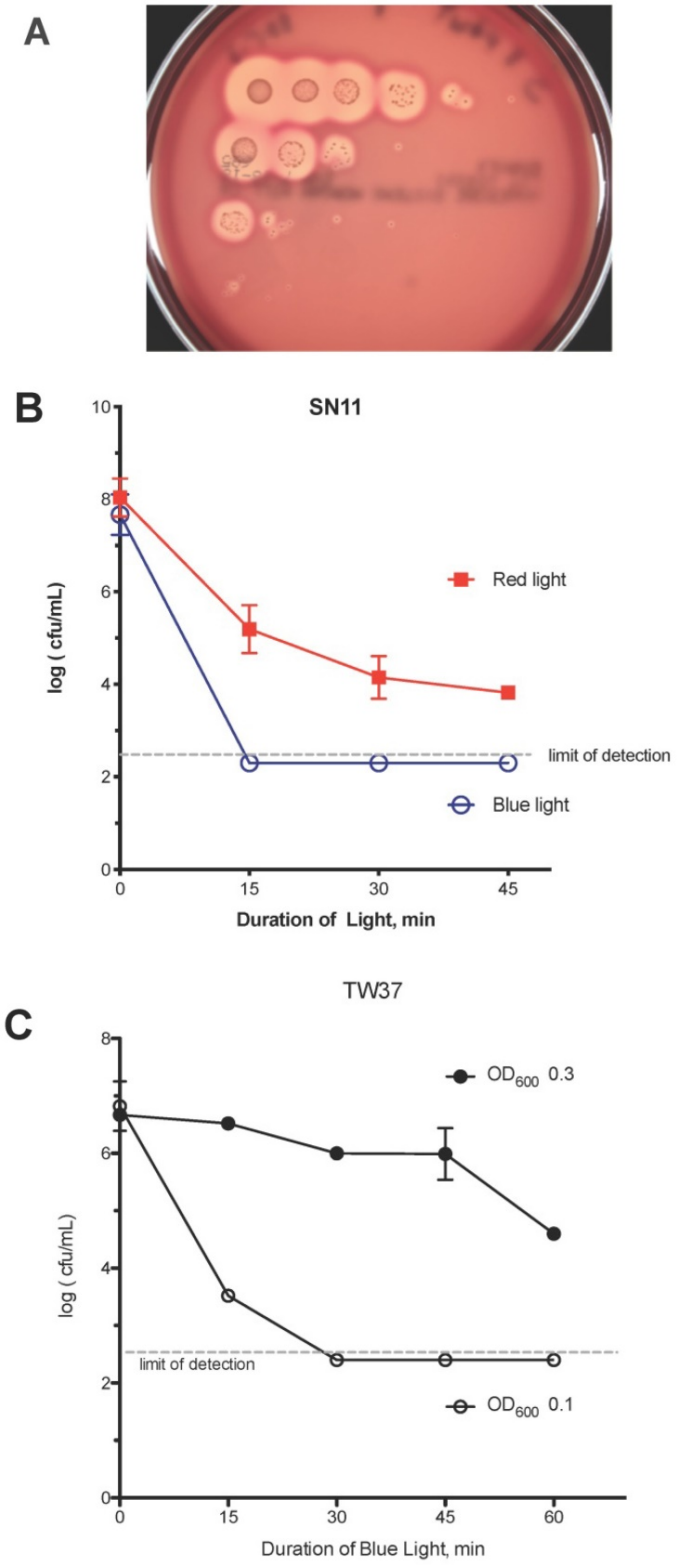
** Methods used for blue light killing of *C. acnes***. (A) Serial 10-fold dilutions of *C. acnes* were performed and plated on Brucella Blood Agar plates to estimate the number of *C. acnes* remaining viable. From top left, row 1 denotes *C. acnes* growth with 0 minutes i.e. no blue light exposure and subsequent rows denote growth after 15, 30 and 45 minutes of blue light exposure respectively. (B) Comparison between red and blue light in their ability to kill *C. acnes* showing that blue light was superior to red light in killing *C. acnes*. (C) Effect of culture turbidity on blue-light-induced killing of *C. acnes* strain TW37.

After 48 hours in anaerobic conditions using GasPak EZ Pouch System (BD, Sparks, MD), the plates were examined and scored for growth (Fig 1A). Bacterial densities were calculated (CFU/mL), converted into a log scale and graphed using GraphPad Prism software (San Diego, CA). The Omnilux light source can be set to emit red light as well as blue light. Blue light was more effective in killing *C. acnes* bacteria than red light, as shown in Fig 1B. For this reason, we focused on blue rather than red light in this study.The importance of adjusting the culture turbidity in response to blue light is reflected by TW37 strain becoming much more resistant to blue light when suspended at a higher turbidity (OD_600_= 0.3) compared to the same strain at an OD_600_ of 0.1 (Fig 1C).

### Blue Light Susceptibility Categories

The following categories were created to classify the susceptibility to blue light based on observed susceptibility patterns: *Highly Sensitive* (*HS*) strains defined as those that were eradicated within 15 minutes. *Sensitive* (*S*) strains demonstrated a ≥3-logfold reduction in bacterial density by 60 min, while *Weakly Sensitive* (WS) strains demonstrated a 1-log to 3-log reduction in CFU/mL, *Resistant* (*R*) strains were defined as those showing a reduction in bacterial counts ≤1 log. Enhancement of the effect of blue light with the addition of photosensitizers was considered substantial if the strains previously *Resistant* to blue light alone became *Sensitive* or* Highly Sensitive* with addition of photosensitizer. The limit of detection was 330 CFU/mL on Brucella Blood Agar plates. Strains were considered eradicated if they fell below the limit of detection. Table 1 summarizes the effect of blue light alone, a combination of blue light with each of the 3 photosensitizers, and the results of antibiotic susceptibility testing to tetracyclines.

### Data Analysis and Statistics

GraphPad Prism software was used to determine statistical differences, either by t-tests or ANOVA analysis, and also to create the graphs shown in the Figures. It was not possible to determine statistical significance for the conditions where bacterial counts were below the limit of detection, because the logarithm of zero is undefined. A p-value less than 0.05 was considered to be statistically significant. Fisher's exact test was used for contingency table calculations.

## Results

### Blue Light Alone

**Figure 2:**
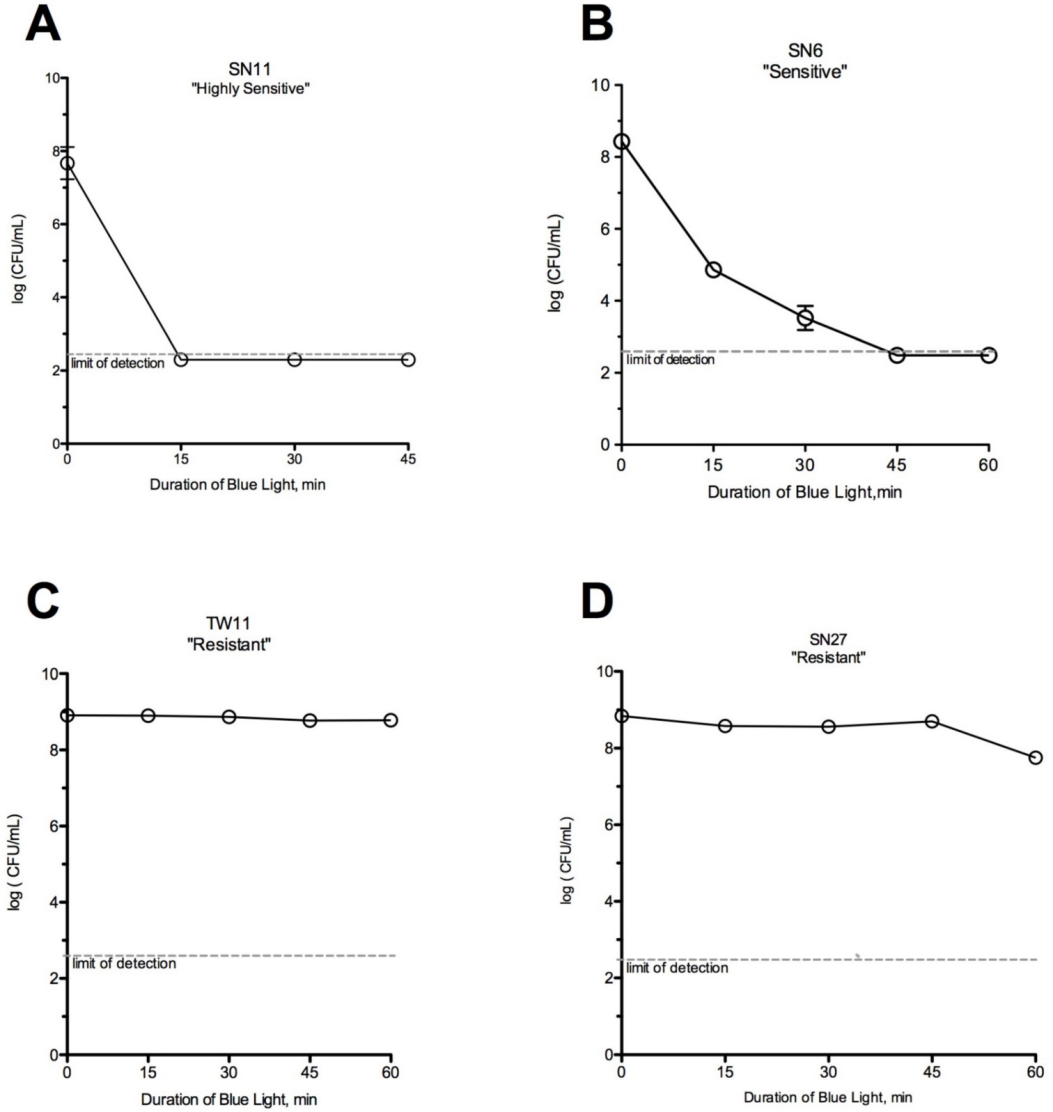
** Susceptibility of *C. acnes* strains to blue light alone.** (A) SN11 was killed by 15 minutes of exposure to blue light alone (*HS*). (B) Strain SN6 required a longer period of exposure to be killed by blue light alone (*S*). Strains (C) TW11 and (D) SN27 were not killed despite 60 minutes of blue light exposure (*R*).

*C. acnes* strains varied in their susceptibility to blue light. Strain SN11 was killed by 15 minutes of exposure to blue light alone (Fig 2A), while other strains required longer periods of exposure (Fig 2B). Two *C. acnes* strains (Figs 2C & 2D) were not killed despite 60 minutes of blue light exposure. Based on the classification system described above, strain SN11 would be classified as *HS*, strain SN6 would be *S,* and strains TW11 and SN27 would be classified as *R*. The majority of *C. acnes* strains demonstrated enough killing by blue light alone to be classified as either *HS* or *S* (13/19, or 68%) (Table 2).

### Blue Light + Photosensitizers

The sensitivity results from testing with blue light alone drove the decision as to which strains would be tested with the addition of photosensitizers. Strains that were *HS* to blue light alone were killed so quickly by light alone that we could not assess any additional effect of photosensitizers, which resulted in 17 of 19 strains remaining for evaluation.

As we accrued data, refined our observations and assessed resources, fluorescein and riboflavin activity proved to have limited killing effect whereas demeclocycline provided very promising results. Fluorescein and riboflavin were tested on 11 strains and demeclocycline on 17 strains (Table 2).

### Blue Light + Fluorescein

**Figure 3:**
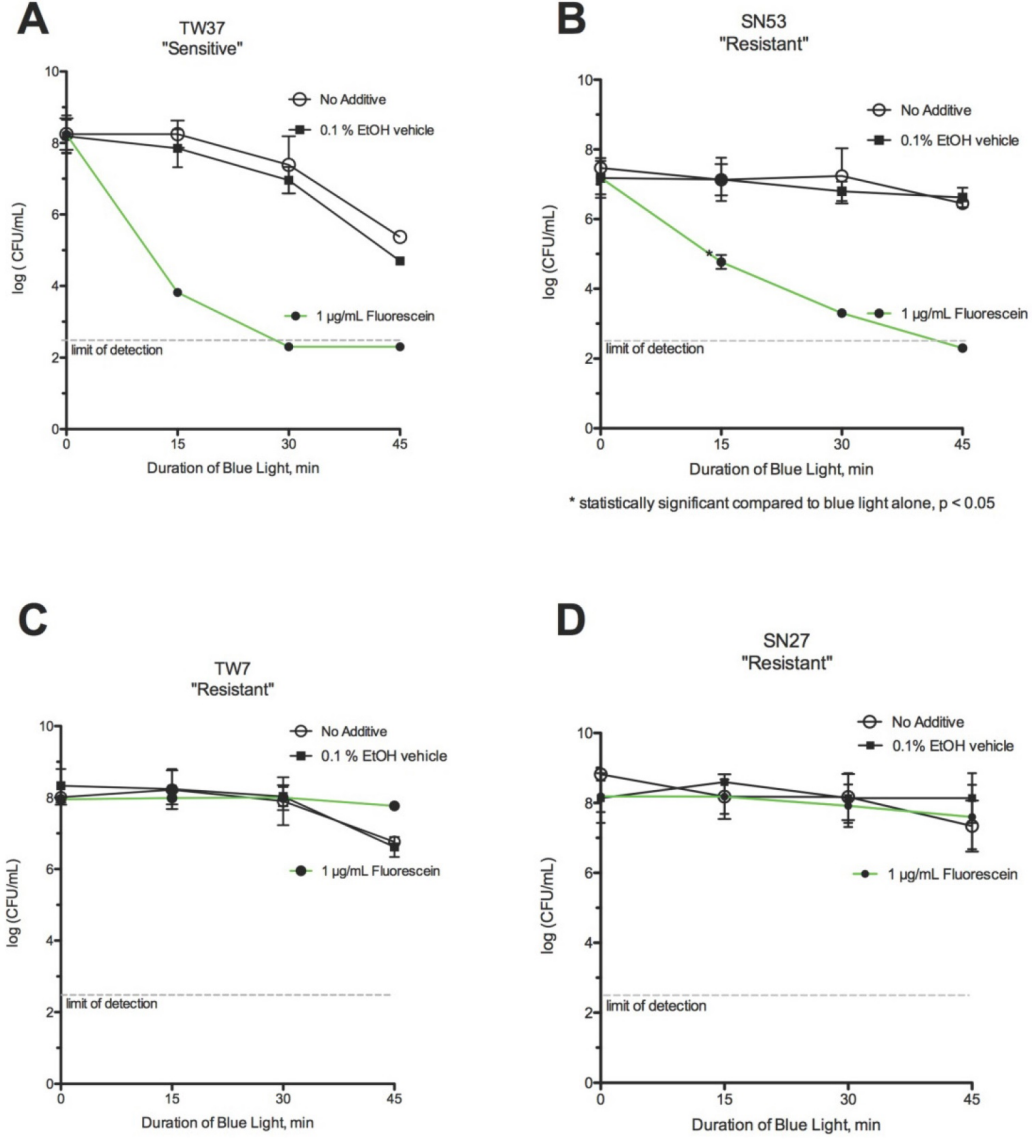
** Effect of fluorescein and blue light.** (A) Strain TW37 was *Sensitive* after 45 min of exposure to blue light alone, but was eradicated after 30 minutes in the presence of fluorescein*.* (B) In the presence of fluorescein, the *Resistant* strain SN53 could be eradicated by blue light following 45 minutes of exposure. Strains (C) TW7 and (D) SN27 remained *Resistant* to the combination of blue light with fluorescein.

Of 11 strains tested, 6 strains were classified as *S* or *HS* to blue light with fluorescein (55%). Fluorescein greatly accelerated the rate of killing for some *C. acnes* strains (Figs 3A-3B), although some strains resisted killing even in the presence of fluorescein (Figs 3C-3D).

### Blue Light + Riboflavin

**Figure 4:**
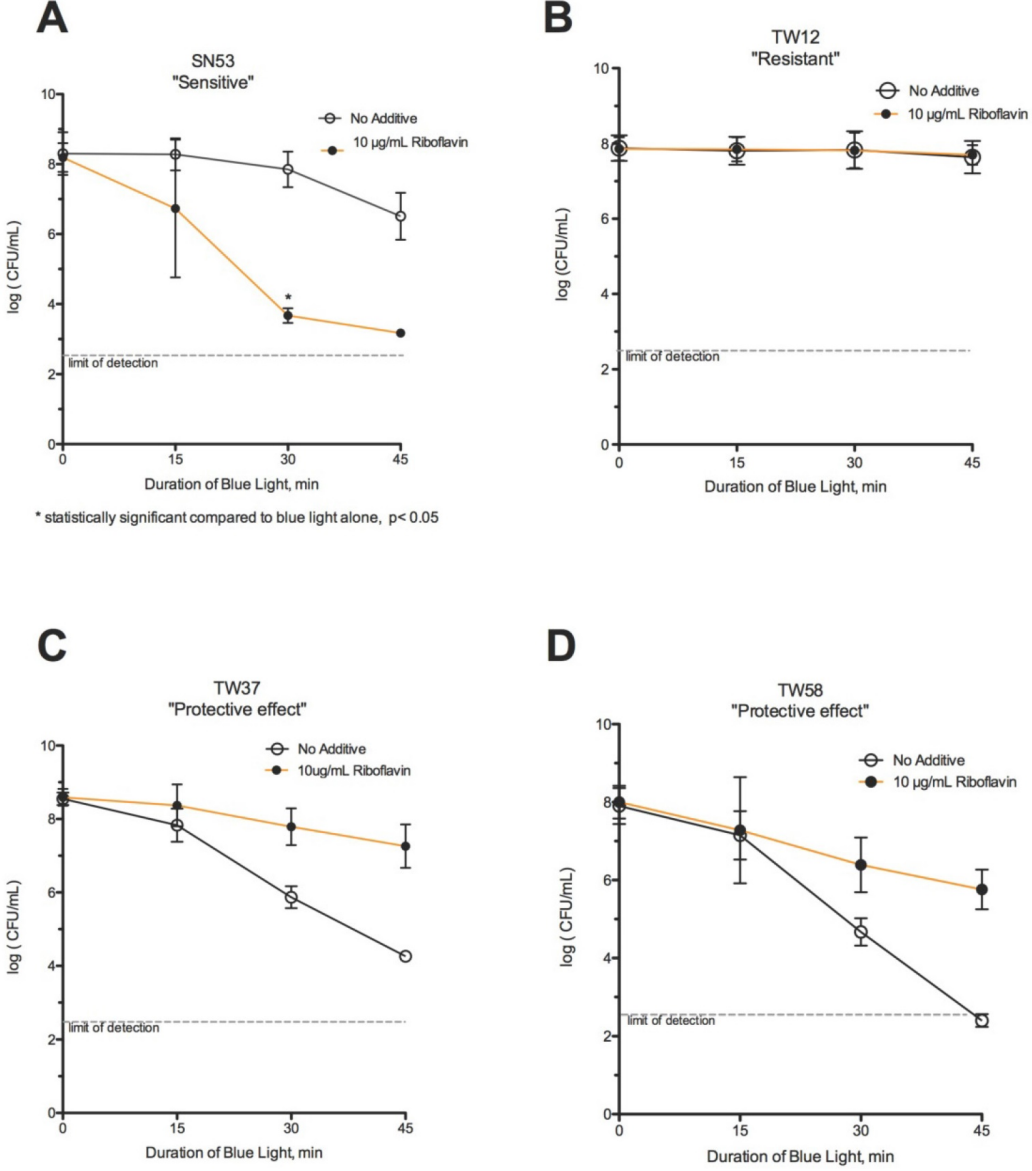
** Effect of riboflavin and blue light.** (A) The combination of blue light with riboflavin enhanced the killing of strain SN53, which statistically significant based on one tailed t-test analysis compared to blue light alone. (B) Riboflavin did not potentiate killing by blue light in strain TW12, a *Resistant* strain. In comparison, riboflavin had a protective effect on strains (C) TW37 and (D) TW58, preventing killing by blue light.

Among the 11 strains tested with riboflavin, 3 showed enhanced killing with riboflavin, while 4 strains showed the paradoxical protection, and 4 showed no significant change compared to blue light alone. Only 3 of 11 strains could be classified as *S* or *HS* to blue light with riboflavin (27%), a net decrease compared to blue light alone. Strain SN53 showed much faster killing in the presence of blue light with riboflavin than with blue light alone (Fig 4A). In contrast, riboflavin failed to potentiate killing in TW 12, a *R* strain (Fig 4B). Riboflavin exerted a protective effect in some strains (Figs 4C-4D), acting as a photo-protectant rather than as a photosensitizer to blue light.

### Blue Light + Demeclocycline

**Figure 5:**
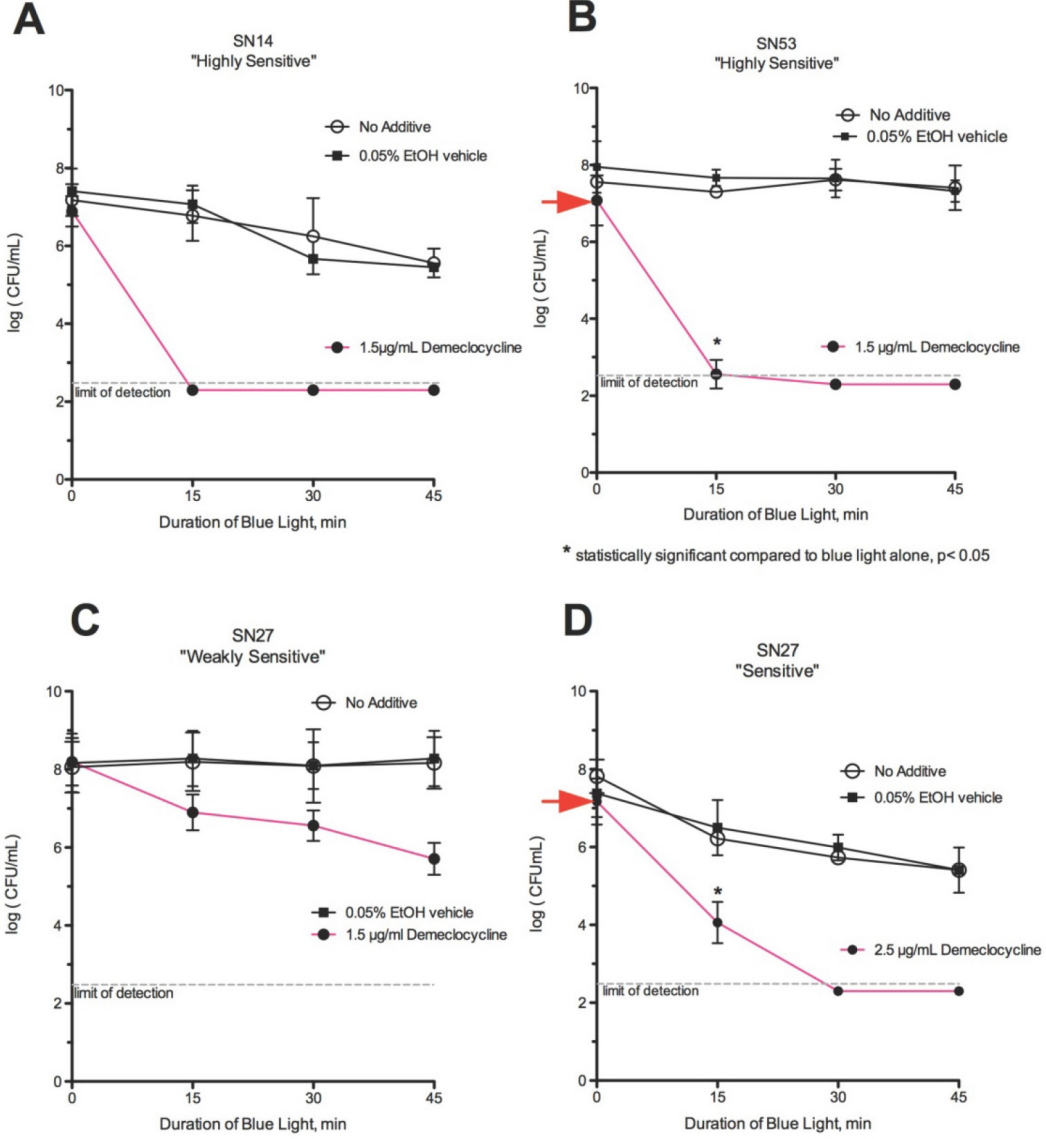
** Effect of demeclocycline and blue light.** (A) Strains SN14 and (B) SN53 became *HS* using blue light with demeclocycline. (C) Strain SN27, our most resistant strain, was *WS* using 1.5 µg/mL of demeclocycline with 45 minutes of blue light exposure. (D) Strain SN27 was *S* using 2.5 µg/mL of demeclocycline and was completely killed in 30 minutes in combination with blue light. *Statistically significant compared to blue light alone.

Demeclocycline enhanced the effect of blue light in 18 of 19 strains (95%). Demeclocycline (1.5 µg/mL) strongly potentiated the effect of blue light in some *C. acnes* strains, rendering them *HS* (Fig 5A-5B). In contrast, demeclocycline (1.5 µg/mL) produced only a modest potentiation of the effect of blue light on strain SN27, a *R* strain, rendering it *WS* (Fig 5C). When the concentration of demeclocycline was increased to 2.5 µg/mL, complete eradication was achieved after 30 minutes of blue light exposure, rendering it *S* (Fig 5D). The effect of killing with blue light and demeclocycline was statistically significant compared to blue light alone (Fig 5B & 5D).

### Demeclocycline Alone (No Photosensitizer)

Demeclocycline alone, without blue light, was observed to have a mild inhibitory effect on growth, with a 0.5-0.7-log reduction in some *C. acnes* strains as shown (Figs. 5B & 5D, y-axis, red arrows). Strains that were *R* to blue light alone became *S* in the presence of blue light with demeclocycline. Strain SN74 was an exception in that conversion occurred from *S* to *WS* in the presence of blue light + demeclocycline.

### Blue Light & Doxycycline Resistance

Examination of Table 1 seemed to indicate that the strains most resistant to the tetracyclines also seemed to be the strains resistant to blue light alone. Table 3 shows a 2 × 2 Contingency Table analysis of the relationship between blue light resistance and doxycycline resistance in our collection of 19 strains. Analysis by both Chi-Squared Test and Fisher's Exact Test showed a significant correlation between blue light resistance and doxycycline resistance, a finding that was unexpected.

## Discussion

*C. acnes* presents something of a paradox to clinicians and microbiologists. Despite being highly sensitive to most antibiotics used for peri-operative prophylaxis 12, 27, *C. acnes* is able to survive and cause PJI 20, 35. This ubiquitous anaerobic, non-motile and non-spore forming Gram-positive bacterium resides in the deep dermis, sebaceous glands, and hair follicles especially in the shoulder region and appears to persist even after commonly used pre-operative skin disinfectants, such as chlorhexidine 20. The dermatology literature contains robust evidence suggesting that blue light therapy may improve acne of the skin caused by *C. acnes* 25, 26. These findings generated the rationale for evaluating the novel application of blue light against a collection of *C. acnes* strains isolated from confirmed periprosthetic shoulder infections.

Our study demonstrated that *C. acnes* strains showed variability in their response to blue light alone, which directed our efforts to further evaluate the utility of photosensitizers. Determination of optimal photosensitizers capable of killing the remaining strains not susceptible to blue light alone provided a considerable challenge. Although attractive due to low toxicity, activity against other bacteria and use in many aspects of medicine, fluorescein did not provide the desired effect as only slightly more than half of the strains were killed when exposed to blue light. Riboflavin has similar attractive qualities to fluorescein as a photosensitizer, but demonstrated a decreased ability to kill *C. acnes* with less than one third of strains killed {Backman, 2014 #64}. Interestingly, about one third of strains showed paradoxical photoprotection in the riboflavin plus blue light condition. In a study evaluating the antibacterial effects *in vitro* on *S. epidermidis* using various riboflavin and UV light protocols, reduction of pathogens appeared to be greater in the less concentrated (0.03%) riboflavin solution than for the higher concentrations {Backman, 2014 #64}.

Porphyrins have been mentioned as photosensitizers and potential endogenous targets of blue and UV light in *C. acnes*, but the supporting data is circumstantial. Ashkenazi et al. showed that *C. acnes* strains showed increased sensitivity to blue light when grown in 5-aminolevulinic acid (5-ALA), the precursor for porphyrin synthesis 21. However, Choi et al. showed that treatment of C*. acnes* with 5-ALA increased the bacterial susceptibility to red light more than blue light 36. This is an interesting but puzzling finding in that porphyrins absorb light intensively in the UV (400-410 nm) and blue light (400-450 nm) regions, and to a lesser extent in the long visible bands, such as orange (~590-635 nm) and red light (~635-700 nm). In addition to porphyrins, endogenous molecules that can absorb blue light include flavins and nicotinamides 21, 36, 37.

Demeclocycline plus blue light was very effective at killing *C. acnes* strains and demonstrated the most substantial results of all photosensitizers. The small decrease in CFU/mL noted at time zero in Fig 5B and 5D is the “dark effect” or “dark toxicity” of demeclocycline, but is dwarfed by demeclocycline's much larger photodynamic effect. Interestingly, blue light plus demeclocycline was able to kill *C. acnes* strains that were resistant to doxycycline and demeclocycline. This observation provides a vivid demonstration of the difference between the antibiotic effect and the photodynamic effect. Additionally, there was a correlation between doxycycline resistance and blue light resistance in that the strains most resistant to tetracyclines also seemed to be resistant to blue light alone. Although this finding is somewhat clouded by the difficulty in determining antibiotic resistance breakpoints for tetracyclines in *C. acnes*, this correlation is reminiscent of our previous finding of a link between hemolytic phenotype and clindamycin resistance 27. This correlation is perplexing as resistance to tetracyclines is often achieved by up-regulation of efflux pumps or by mutations in ribosomal RNA, neither of which suggest an obvious pathway that would be sensitive to blue light.

There are limitations to this study and many variables that were not evaluated. Our study does not reveal the identity of the endogenous photosensitive molecules in those strains sensitive to blue light alone or those that become *HS* with the addition of photosensitizers. The molecules that act as photoreceptors for blue light in *C. acnes* are still not conclusively known, and further research is needed to understand the mechanism of action. Additionally, our study does not fully replicate the clinical application of blue light. We did not simulate penetration of the blue light into the deep dermal layers of the skin, which would be needed to eradicate *C. acnes* clinically. In our study, bacterial suspensions were exposed to blue light in air, whereas exposure in a purely anaerobic environment might alter the results. These limitations may influence the translational clinical applications of our *in vitro* findings. Further research is required to determine if blue light is a clinically relevant treatment modality against *C. acnes*. In addition, the association between blue light resistance and antibiotic resistance (Table 3) needs to be confirmed using a larger number of strains.

Blue light plus photosensitizers killed *C. acnes* from periprosthetic shoulder infections *in vitro*, with demeclocycline having the most pronounced effect and riboflavin demonstrating a photoprotective effect in one third of strains. Future work will focus on refining optimal photosensitizers and variables of blue light exposure that can translate into the development of *in vivo* models.

## Supplementary Material

Supplementary figures and tables.

## Figures and Tables

**Table 1:** Susceptibility of *C. acnes* Strains to Blue Light, Blue Light + Photosensitizers, and Tetracycline Antibiotics

Strain	Effect of Blue Light Alone ^a^	Effect of Blue Light Plus Sensitizers			MIC to Doxycycline, mg/L , *^b^*	MIC to Demeclo-cycline, mg/L, ^c^	References, Comments
		Fluorescein	Riboflavin	Demeclo-cycline			
SN6	S	HS	WS	HS	0.047	≤ 0.5	Ref. 12 for SN strains
SN9	S	HS	WS	HS	0.75	1.0	
SN11	HS	--	--	--	0.125	1-2	
SN14	R	WS	WS	HS	0.047	≤ 0.5	
SN27	R	R	S	S^d^	1.5	> 4	
SN53	R	S	S	HS	0.38	1	
SN71	S	n.d.	n.d.	S	.125	1.0	
SN73	S	n.d.	n.d.	HS	0.023	≤ 0.5	
SN74	S	n.d.	n.d.	WS	0.047	≤ 0.5	
SN80	S	n.d.	n.d.	S	0.023	≤ 0.5	
TW6	S	n.d.	n.d.	HS	0.064	≤ 0.5	Ref. 27 for TW strains
TW7	R	R	WS	S	0.094	≤ 0.5	
TW10	S	n.d.	n.d.	HS	0.094	≤ 0.5	
TW11	R	S	R	S	32	> 4	
TW12	R	R	R	S	32	> 4	
TW37	S	S	WS	S	0.047	≤ 0.5	
TW38	HS	--	--	--	0.094	≤ 0.5	
TW58	S	R	WS	HS	0.094	≤ 0.5	
TW64	R	S	HS	HS	0.25	≤ 0.5	Fastest growing strain

^a^, Categories for sensitivity to light, with or without photosensitizers, were as explained in Materials and Methods. --, unable to test additive effect of photosensitizers because strain was already Highly Sensitive (HS). n.d., not done; ^b^, MIC determined using MIC Strips; ^c^, MIC determined by Agar Dilution on BHI + glucose; ^d^, a concentration of 2.5 µg/mL of demeclocycline was used to achieve this result.

**Table 2:** Summary of *C. acnes* killing with blue light alone vs blue light + photosensitizers

Condition	Vehicle	Concentration	Strains Highly-Sensitive or Sensitive
Blue light	None	-	13/19 (68%)
Fluorescein	0.1% EtOH	1 µg/mL	6/11 (55%)
Riboflavin	H_2_O	10 µg/mL	3/11 (27%),
Demeclocycline	0.05% EtOH	1.5-2.5 µg/mL	16/17 (94 %)

**Table 3:** Two x Two Contingency Table for Doxycycline Resistance and Blue Light Resistance

Categories: Susceptibility to Blue Light	Strains with a Doxycycline MIC of ≤ 0.75 µg/mL *	Strains with a Doxycycline MIC of ≥ 1.0 µg/mL ^#^	p-value
*Weakly Sensitive, Sensitive,* or *Highly Sensitive*	12	0	0.036
*Resistant*	4	3	

*presumed susceptible; #presumed resistant, based on older references. The most recent versions of Breakpoint tables from the Clinical Laboratory Standards Institute (CLSI) and the European Committee on Antimicrobial Susceptibility Testing (EUCAST) omit interpretations for the tetracyclines in *C. acnes.*
