# Supplementary material for: Effect of Blue Light and Photosensitizers on Cutibacterium acnes on Shoulder Periprosthetic Joint Infection Isolates
Source: J Bone Jt Infect. 2020 Jun 20;5(4):187–97. doi: 10.7150/jbji.46199 (PMC7358969; doi:10.7150/jbji.46199)
Supplement: Supplementary file 1 — Supplementary figures and tables. [file jbjiv05p0187s1.pdf]

Flow Diagram for Blue Light Experiments

A

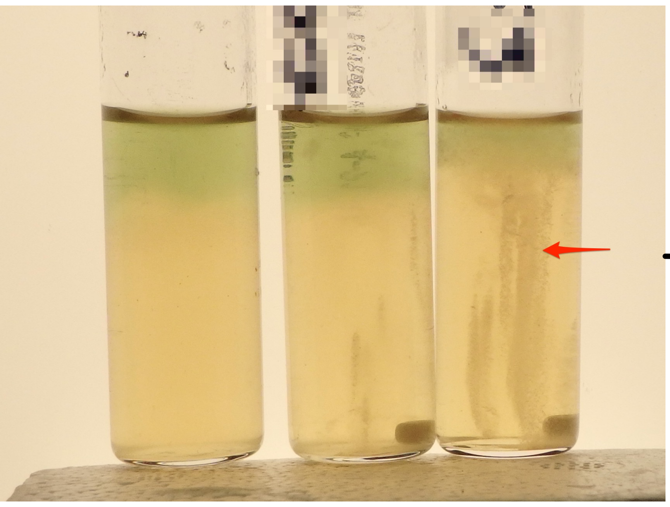

Grow *C. acnes* strains in thioglycollate medium until visible growth occurs, as shown by the red arrow in the tube on the right

B

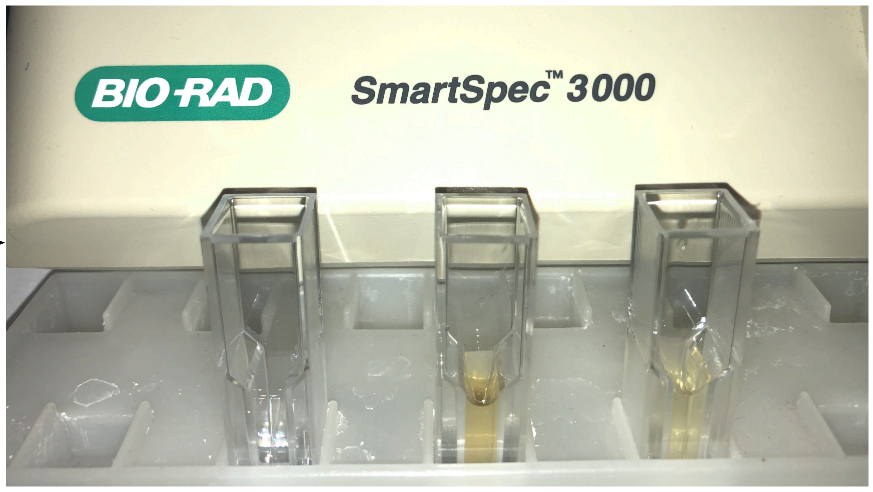

Dilute in sterile saline and adjust OD600 to 0.1 to 0.15 using spectro-photometer

Warm Suspension to 37 ° C

C

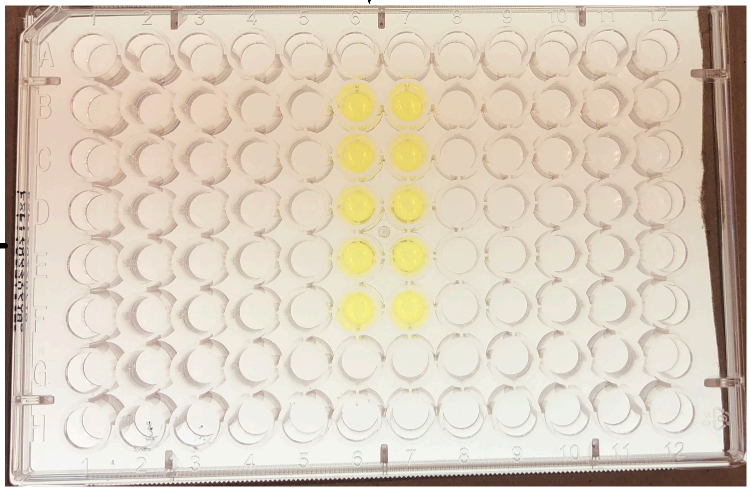

Place 250 µl into the center wells of a 96 well plate; in a real experiment, only some of the wells would contain the photosensitizer, such as fluorescein (yellow color) shown here.

D

37 ° incu-bator

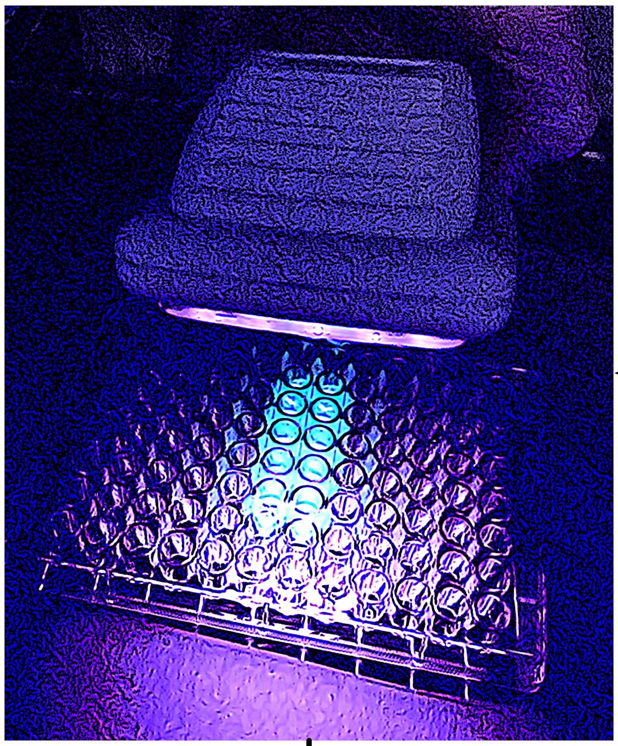

Irradiate with blue light; the light is placed directly on top of the 96 well plate. New samples are added at 15 min intervals for time courses.

E

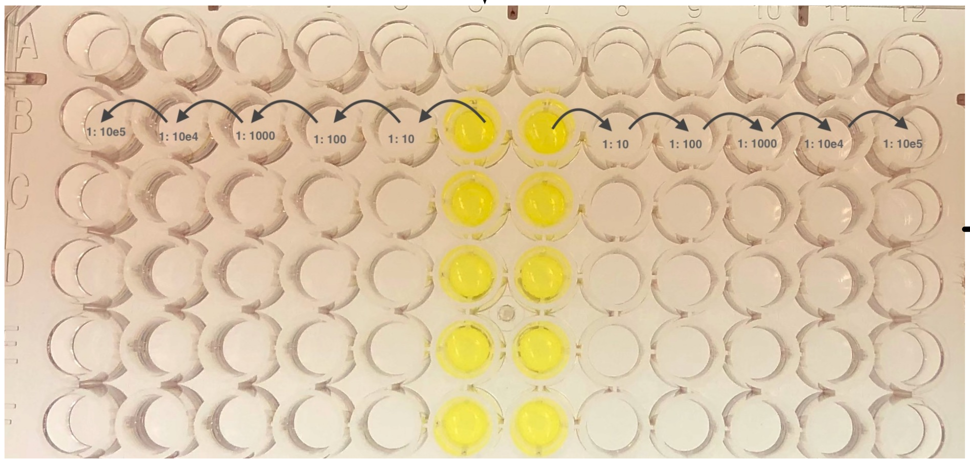

After adding the “Dark Controls,” a multi-channel pipettor is used to perform serial, 10-fold dilutions on the irradiated and control suspensions.

F

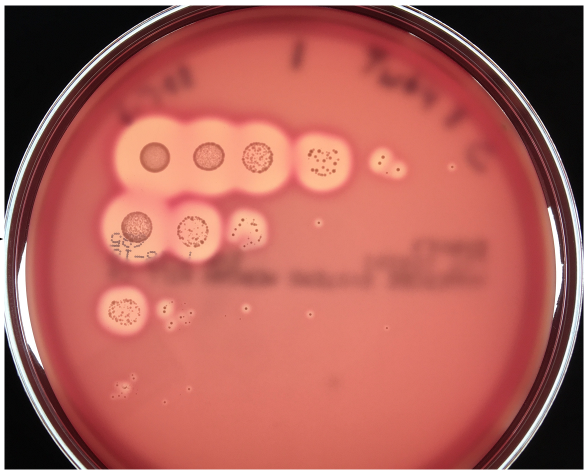

The diluted and undiluted suspensions are spotted onto Brucella Blood Agar and incubated anaerobically, then counted.
